# Crockett’s Gynecology

**Published:** 1900-05

**Authors:** 


CROCKETT'S GYNECOLOGY. A Pocket Text-Book on Diseases of Women. By MONTGOMERY A. CROCKETT, A. B., M. D., Adjunct Professor of Obstetrics and Clinical Gynecology, Medical Department of the University of Buffalo, N. Y. In one handsome 12mo volume of 368 pages, with 107 illustrations. Philadelphia and New York: Lea Brothers & Co. 1900. [Price, Cloth, $1.50, net; flexible red leather, $2.00, net.]

The reviewer set about a task which sometimes is irksome with the feeling that a Buffalo author must be given more than the usual attention bestowed on a book for review. So well written is this volume, however, that the task soon became a pleasure.

There are many points especially worthy of mention. Chapter two on local treatment describes but three methods of procedure, the prolonged hot vaginal douche, the tampon, and intrauterine applications, and of the last the author very justly remarks: "There are a few cases of uncomplicated metritis which may be benefited by local applications to the endometrium; but they are so few that womankind as a whole would be the gainer were topical applications within the uterus utterly abolished, except in connection with the operation of dilating and curetting."

The third chapter deals with gynecologic operations in general. The author has this to say apropos of suture material: "It is hard to understand why surgeons persist in using buried nonabsorbable ligatures and sutures when the formaldehyde gut is so easily prepared and possesses the ideal features of sterility, strength and absorbability." Again, "there is no excuse for the intraäbdominal use of any other material than formaldehyde catgut, and the surgeon who exposes his patients to the dangers which follow the use of silk sutures and ligatures, should be held responsible for untoward results." Many surgeons will say "amen" to this, and not a few patients might. It is interesting to note that the editor of the series takes exception to Dr. Crockett's strong statement. And so do a good many others with far more experience than Dr. Crockett; nevertheless there is much in his statement that may prove worthy of consideration.

Some operators may consider that Dr. Crockett uses saline solution in intraäbdominal operations in a somewhat homeopathic manner, when he recommends that it be poured into the pelvis in small quantities, swabbed about and removed; and further, that it is a good plan to leave in the abdomen a pint or two of the solution.

Concerning hand disinfection, the disinfection of the vagina, drainage, in short all matters relating to preparation of patient and operator, the author sets forth the latest and most generally accepted methods.

The greater part of the book deals naturally with the various pathologic conditions to which the female genital organs are prone. Great originality in writing on such subjects is not possible nor, where one is addressing students, desirable. The author has aimed rather to make the work representative, and in this he has succeeded so well, and has presented his facts with such lucidity, that one would need to go far to find a more readable and altogether valuable minor volume on gynecology. If Dr. Crockett has a fault it is that of being too positive, a failing not uncommon with teachers, and not at all serious. Indeed, it is probably the author's ability as a teacher of medicine, an ability of more than local note, which enables him to present a text-book so admirably adapted to the minds of the student and young practitioner.

The preface makes acknowledgement of the writer's indebtedness to Dr. M. D. Mann for many valuable criticisms and suggestions.

This volume is printed and bound in uniformity with Leas' pocket text-book series. M. J. F.

